# Tissue-engineered fibrillar fibronectin matrices are not only lovely, but also functional for regenerative medicines and *in vitro* model systems

**DOI:** 10.1016/j.bbiosy.2024.100104

**Published:** 2024-12-02

**Authors:** Seungkuk Ahn

**Affiliations:** UCD Charles Institute of Dermatology, School of Medicine, University College Dublin, Belfield, Dublin 4 - D04V1W8, Ireland

**Keywords:** Fibrillar fibronectin, Extracellular matrix, Integrin, Mechanobiology, Fibrillogenesis, Tissue engineering, Biomaterials

## Abstract

Fibronectin is an ubiquitous extracellular matrix protein which comprises fibrous three-dimensional microenvironments in native tissues. Although its importance and fibrillogenesis *in vivo* has been considerably investigated, yet current *in vitro* tissue engineering platforms for fibrillar fibronectin pose major drawbacks such as low scalability, applicability, and reproducibility. Due to such platform limitations, understanding of spatiotemporal mechanobiology between cells and fibrillar fibronectin matrices largely remains unexplored. This article briefly underlines current tissue-engineering platforms and mechanobiological understanding of fibrillar fibronectin as well as suggests potential directions in future fibronectin researches.

## Highlights

- Fibrillar fibronectin serves as a physiological and regenerative building block in tissues.
- Tissue engineering three-dimensional fibrillar fibronectin matrices is currently challenging.
- Fibronectin-driven mechanobiology needs to further take account various biological and temporal scales.
- Future fibronectin research should pursue to improve the engineering platform and mechanobiological understanding of fibrillar fibronectin matrices in three-dimensional context.

## Fibrillar fibronectin as a functional and regenerative building block in native tissues

**Fig. 1:**
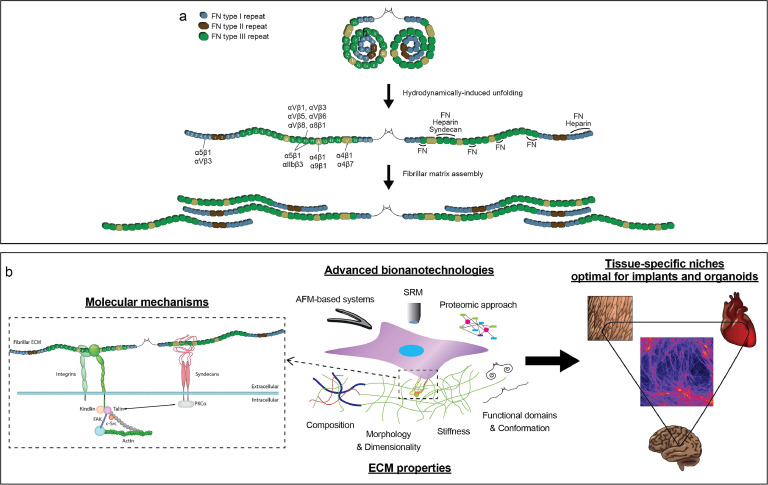
Fibrillar fibronectin (FN) serves as a regenerative building block to ultimately design tissue-specific niches for advanced implants and *in vitro* culture platforms. a. Conformational changes from a globular form to a fibrillar (often called as cellular or unfolded) form during FN fibrillogenesis further uncover protein and cell binding sites while being self-assembled into fibrillar matrices. b. Schematic summary of the proposed future direction of FN researches to deepen fibrillar FN engineering platforms and FN-driven mechanobiology towards development of tissue-specific niches.

In tissues, extracellular matrix (ECM) is a network of proteins and other components which provide compositional and structural supports for distinctive cellular behaviors and functions. ECMs pose three-dimensional (3D) fibrous structures where cells attach and grow within. Fibronectin (FN) is a major fibrillar ECM component crucial for tissue development and repair [1]. FN is comprised of type I, II, and III repeating units which have self-assembly, cell-binding (*e.g.,* integrin), or protein (*e.g.,* collagen) binding domains (Fig. 1**a**). Synthesized by hepatocytes, soluble globular FN circulates in the bloodstream and recruited during tissue repair. Globular FN is an inactive form which lacks EIIIA, EIIIB, and variable (or V) regions compared to fibrillar FN [2].

Once delivered to tissues, globular FN is polymerized into fibers to form intricate ECM networks. The fibrillogenesis of FN are mainly driven by cells via integrin binding and contractility-mediated unfolding/self-assembly [1]. Firstly, globular FN binds to integrins (*e.g.,* α5β1 integrins) and then undergo conformational changes (from a compact to an extended form) by traction forces generated by cellular tension. Conformational changes of globular FN unfold its modules to uncover cryptic functional domains buried in globular FN that accelerate FN-FN and FN-other ECM interaction as well as regulate cell behaviors and fates over its lifespan. This is the reason why fibrillar FN, but not globular FN (or non-fibrillar, plasma FN), is a functional and structural form of FN in tissues as well as why fibrillar FN is often called as ‘cellular FN’ in the field. Finally, unfolded FN and resulting intermolecular FN-FN interactions further progress fibrollogenesis toward crosslinked aggregates of FN with a high-molecular weight and fibrillar form [2]. It was speculated that unfolded FN molecules significantly overlap each other by being staggered and linearly arranged and such N-terminal overlap among neighbouring FN molecules could be strengthened by the inter-molecular interactions including FNI and FNIII_1–5_ domains [3]. FN is also a core integrin binding protein which orchestrates integrin-mediated cell responses including adhesion, migration, and differentiation.

FN cooperatively work with other ECM proteins (*e.g.,* laminin and collagen) to build complex tissue microenvironments by utilizing protein binding domains and electrostatic interactions. For instance, fibrillar FN acts as a key nucleator of collagen I fibrillogenesis and microtissue formation which can be accelerated during macromolecular crowding [4,5]. Full-length globular FN (but not FN fragments) embedded within collagen hydrogel was able to direct cell migration, differentiation, and tissue morphogenesis, suggesting that potential fibrollogenesis of full-length globular FN and resulting fibrillar FN together with fibrillar collagen matrices play a key role in tissue development, homeostasis, and repair [6].

Lastly, fibrillar FN is greatly related to improved tissue regeneration possibly due to accelerated cell adhesion, growth, and new ECM deposition activated by the fibrillar FN-driven mechanosignaling. In particular, fibrillar FN promotes platelet adhesion and thrombus growth via α5β1 and αIIbβ3 integrins and EDA domains which are mostly hidden in globular FN [7]. The recent studies led by the author showed that fibroblasts strengthen their adhesion, migration, and proliferation in response to fibrillar FN compared to globular FN via α5β1 integrins and syndecan-4 crosstalk. Compared to globular FN, fibrillar FN also boosts cancer cell invasion, proliferation, and expansion [8,9]. Finally, fibrillar FN poses a potential to facilitate organoid culture, maturation, and morphogenesis [10,11]. These studies highlight how fibrillar FN as an active FN form provides strong potential for tissue engineering and regenerative medicine.

## Challenge: *in vitro* tissue-engineering 3D fibrillar fibronectin matrices instead of 2D fibronectin substrates

Over the last decade, researchers have strived to understand the impact of FN properties and the spatial sensing of FN by cells *in vitro*. However, the majority of researches was conducted on very simplified FN models that use peptidomimetics (*e.g.*, arginine-glycine-aspartic acid (RGD) containing small peptides), FN fragments or globular FN developed by biological methods and attached them to two-dimensional (2D) substrates [12]. These simplified FN models lack of three dimensionality, fibrillar architecture, and functional domains that determine diverse biophysical and structural cues of native fibrillar FN matrices and the cellular response to these matrices, resulting in disparity between different *in vitro* and *in vivo* studies. Particularly, our recent studies affirm that cells (*e.g.,* fibroblasts, neurons, epithelial cells, and organoids) recognize and respond differently to fibrillar FN matrices compared to globular FN coatings across various temporal (from a few seconds to several weeks) and biological scales (from single cell adhesion to tissue morphogenesis) [10,13]. Hence, developing fibrillar FN matrices has emerged as an important mission in the field of tissue engineering to develop physiologically-relevant and regenerative biomaterials. So far, beyond the simplified 2D FN models from the mainstream, there were two main cell-free strategies to produce fibrillar FN by 1) chemical or 2) physical methods. Firstly, the chemical methods employ negatively charged polymers (poly sulfuric acid or poly(ethyl acrylate)) or surfactants (polyvinyl alcohol) to modulate surface charge and tension to trigger FN unfolding and self-assembly processes by mimicking surface interaction from FN-integrin during *in vivo* fibrillogenesis [14].

Another approach is physical methods to produce fibrillar FN. The physical methods use interfacial molecular forces (*e.g.,* shear force or surface energy) to induce fibrillar FN formation by mimicking actomyosin contractility-driven FN unfolding and self-assembly *in vivo*. The oldest and most widely used approach is to manually pull FN solutions with a pipet tip which generates mild shear forces between air and solution interfaces to include FN unfolding and produce a single FN fiber [15]. Furthermore, protein-surface interactions such as surface energy can be tuned to form FN nanofabrics by absorbing globular FN and releasing the absorbed FN from a thermosensitive polymer surface to the solution medium [16]. The released FN nanofabrics are actually composed of globular FN, but can be stretched further from globular to unfolded fibrillar FN forms. The FN nanofabrics are free-standing and thus pose a great potential as a cell culture platform and tissue implant. Although both chemical and physical methods mentioned above enabled to induce unfolding and fibrillar assembly of globular FN, they are still limited to 2D structure, poor throughput, poor porosity, low transparency, low reproducibility, and difficulty to scale up. There is thus a great need for developing an easily-applicable platform to engineer 3D fibrillar FN matrices and deciphering their roles in determining cell responses.

Recent studies investigated by the author and others revealed promising platforms to engineer 3D fibrillar FN matrices. In these methods, shear forces as a physical method were applied to unfold globular FN solution and promote self-assembly and aggregates of FN fibrils into 3D fibrillar FN matrices by employing rotary jet spinning or simply rotating micro-porous substrates in FN solution [10,13,17]. The rotary jet spinning method has a high throughput and does not need any support material, while requires a special machine and strong acid (*e.g.,* hexafluoroisopropanol) as a solvent [17,18]. On the other hand, the simple rotation method using hydrodynamic shear forces is easily applicable to any laboratory and produces 3D fibrillar FN from physiological medium, while necessitates a porous support material (which can be 3D-printed or electro-spun in a large batch) and has lower throughput than the rotary jet spinning method [8,10,11,13,19].

Once 2D and 3D fibrillar FN matrices are produced, their stability has to be accounted for their use in tissue engineering applications. FN fibrillogenesis intrinsically induces insoluble fibrillar FN formation after unfolding and self-assembly compared to soluble globular FN. In fact, recent studies showed that hydrodynamic force-induced 3D fibrillar FN matrices were stable up to 210 days for brain organoid culture at physiological pH and temperature without any crosslinking [11]. On the other hand, rotary jet spun FN fibers without crosslinking were degraded within 6 days when implanted to *in vivo* mouse wound models [17]. To address potentially rapid degradation of engineered fibrillar FN matrices by plethora of cells and enzymes for long-term *in vivo* and clinical applications, enhancement of engineered fibrillar FN stability needs to be explored. For instance, our recent studies applied common chemical crosslinkers such as paraformaldehyde or methanol to increase stability of engineered fibrillar FN matrices [13]. More robust crosslinking followed by sophisticated chemical modifications (*e.g.,* methacrylation or biotinylation) could enhance stability of FN-FN (for fibrillar FN only matrices) and FN-other ECM components (for FN-based multicomponent matrices). When applying such chemical crosslinking and modification methods, non-reacted chemical residues and their potential toxicity should be carefully monitored which could negatively affect the regenerative efficacy of fibrillar FN matrices. Collectively, there are still rooms to be improved for the above-mentioned state-of-art platforms.

## Challenge: understanding fibronectin-initiated integrin mechanobiology

The dynamic regulation of adhesion establishment and cellular responses to continuously changing ECM parameters across different temporal and biological scales requires cell receptors (named as integrins) to dynamically bind to and unbind from ECM ligands [20]. Integrins are heterodimers composed of α and β subunits. In particular, α5β1 and αV integrins are known as main integrins to recognize FN to date [1]. Their ectodomains (or heads) in the extracellular region bind to ECM ligands, while their tails in the intracellular region transfer signaling from ECM ligands to intracellular molecules such as actin. These integrin-mediated signaling pathways are triggered by adhesion initiation, are constantly changing, and continue to orchestrate cellular responses over cell life span in response to ECM cues such as composition, stiffness, fibrous structure, and dimensionality.

In the view of FN mechanobiology, globular and fibrillar FN interact differently with integrins and other ECM components. It is well established that unfolding of globular FN to fibrillar FN exposes cryptic sites including integrin-binding and ECM-binding sites [1]. For instance, the EDA and EDB domains are exclusively available in fibrillar FN and provide unique integrin-binding sites (*e.g.,* α4β1, α4β7, α9β1 integrins), whilst enhancing accessibility to adjacent integrin-binding sites such as FNIII_10_ and IIICS which further promotes α5β1 and αV-class integrins compared to globular FN [2]. Similarly, the FNI domains in the N-terminal of FN are buried in globular FN and uncovered during fibrillogenesis. The FNI domains exclusive in fibrillar FN offer additional α5β1, α3β1, αVβ3 integrin-binding sites on top of FNIII_9–10_ or RGD repeats available in both globular and fibrillar FN [2]. Thanks to these extra integrin-binding sites, fibrillar FN can promote cell adhesion, migration, proliferation, differentiation, tissue morphogenesis, and tissue regeneration by triggering integrin-mediated mechanosignaling faster than globular FN [10,11,13,17]. Regarding interaction of FN with other ECM components, FN poses ECM binding sites for fibrin, heparin, tenascin, collagen, and gelatin in FNI and FNIII_12–14_. These ECM binding sites are less accessible in globular FN and become exposed in fibrillar FN to facilitate FN-other ECM binding to optimally reconstruct the complex fibrillar ECM microenvironment in tissues [10]. These underline that fibrillar FN offers strengthened integrin binding and other molecular interaction which is more physiologically relevant for tissue restoration.

Current *in vitro* FN researches in relation to integrin biology mainly focus on the impact of their properties on matured cell adhesion, migration, or its long-term impacts on cell behaviors (*e.g.*, wound healing and differentiation) in 2D contexts. For instance, globular FN or RGD coatings were shown to enhance adhesion maturation, migration, and differentiation via specific integrin signal activation (*e.g.,* α5β1 integrins) and in turn corresponding intracellular signaling molecules such as kindlin, talin, actin, myosin, and paxillin [12,21]. There are yet discrepancies about which integrins accelerate or deaccelerate certain behaviors of different cell types in response to differently engineered FN substrates. Therefore, the initial steps of cell adhesion in response to the FN cues and the progression from adhesion initiation to long-term responses (*e.g.,* tissue regeneration) are neglected albeit being majorly critical in various processes such as immune responses and metastasis *in vivo*. Accordingly, this illustrates the necessity to decipher how integrins initiate and regulate adhesion to complex 3D environments, how this determines initial cellular responses and intracellular signals, how the single cell-level sensing transitions into the tissue-level sensing at a later stage, and how to adopt the intrinsic integrin crosstalk between a single cell, tissue, and ECM cues to design tissue-specific regenerative niche for controlling cellular behaviors.

## Future perspectives

Fibrillar FN poses a significant potential as functional and regenerative building blocks for development of biomimetic materials in tissue engineering and regenerative medicine. The recent studies showed that, significantly different from globular FN coatings, fibrillar FN matrices could activate integrin-mediated signalling cascades to accelerate cellular responses in potentially more physiologically-relevant manners. However, there is still room for improvement of engineering fibrillar FN matrices and understanding their mechanobiology.

A first step should be to optimize an engineering platform to produce 3D fibrillar FN matrices in a more scalable, tunable, and easily applicable manner. Following the similar development rationale of other ECMs such as collagens, various engineering techniques (*e.g.,* 3D printing or fiber spinning) should be rigorously explored to scale up manufacturing of fibrillar FN matrices. Furthermore, how fibrillar FN co-assembles with other core ECM components has to be investigated by employing advanced bionanotechnologies such as atomic force microscopy, high resolution microscopy, and proteomic analysis (Fig. 1**b**). This will provide a better spatiotemporal understanding for the role of fibrillar FN in maintaining and adjusting the heterogeneity of native ECM microenvironments. Finally, we should explore how cells employ integrin-mediated cascades to mechanosense fibrillar FN alone and in presence of other ECM components across different temporal and biological scales. Whether such fibrillar FN mechanosensing is dependent on cell type, structure, time, or co-existing ECM components should be assessed. The last step is then to apply such 3D fibrillar FN matrices for more clinically-relevant applications. For example, 3D fibrillar FN matrices can serve as a regenerative implant to promote various tissue repairs due to its universal presence throughout our body. Moreover, 3D fibrillar FN matrices can be applied to cancer researches. Deposition of fibrillar FN is greatly high in tumour tissues and thereby engineered 3D fibrillar FN matrices can be employed to efficiently expand tumour cells for promoting development of tumour cell-based vaccines as a part of cancer immunotherapies [8,9]. Altogether, these new information will contribute to establish tissue-specific niches, based on fibrillar FN as a building block, which are more clinically relevant and optimal for advanced implants and *in vitro* model systems like organoids.

## CRediT authorship contribution statement

**Seungkuk Ahn:** Writing – review & editing, Writing – original draft, Conceptualization.

## Declaration of competing interest

The authors declare that they have no known competing financial interests or personal relationships that could have appeared to influence the work reported in this paper.

## Data Availability

No data was used for the research described in the article.
